# Cardiac index-guided therapy to maintain optimised postinduction cardiac index in high-risk patients having major open abdominal surgery: the multicentre randomised iPEGASUS trial

**DOI:** 10.1016/j.bja.2024.03.040

**Published:** 2024-05-26

**Authors:** Sandra Funcke, Götz Schmidt, Alina Bergholz, Pilar Argente Navarro, Gonzalo Azparren Cabezón, Silvia Barbero-Espinosa, Oscar Diaz-Cambronero, Fabian Edinger, Nuria García-Gregorio, Marit Habicher, Gerd Klinkmann, Christian Koch, Alina Kröker, Thomas Mencke, Victoria Moral García, Amelie Zitzmann, Susanne Lezius, Amra Pepić, Daniel I. Sessler, Michael Sander, Sebastian A. Haas, Daniel A. Reuter, Bernd Saugel

**Affiliations:** 1 Department of Anesthesiology, Center of Anesthesiology and Intensive Care Medicine, University Medical Center Hamburg-Eppendorf, Hamburg, Germany; 2 Department of Anesthesiology, Operative Intensive Care and Pain Therapy, Justus-Liebig-University Giessen, Giessen, Germany; 3 Department of Anesthesiology, Perioperative Medicine Research Group, Hospital Universitari i Politécnic La Fe, Valencia, Spain; 4 Department of Anesthesia and Pain Management, Hospital de la Santa Creu i Sant Pau, Barcelona, Spain; 5 Department of Anaesthesiology, Intensive Care Medicine and Pain Therapy, University Medical Centre of Rostock, Rostock, Germany; 6 Fraunhofer Institute for Cell Therapy and Immunology, Department of Extracorporeal Therapy Systems, Rostock, Germany; 7 Institute of Medical Biometry and Epidemiology, University Medical Center Hamburg-Eppendorf, Hamburg, Germany; 8 Outcomes Research Consortium, Department of Anesthesiology, Cleveland Clinic, Cleveland, OH, USA; 9 Outcomes Research Consortium, Cleveland, OH, USA

**Keywords:** anaesthesia, cardiac output, cardiovascular dynamics, haemodynamic monitoring, individualised, morbidity, mortality, randomised controlled trial

## Abstract

**Background:**

It is unclear whether optimising intraoperative cardiac index can reduce postoperative complications. We tested the hypothesis that maintaining optimised postinduction cardiac index during and for the first 8 h after surgery reduces the incidence of a composite outcome of complications within 28 days after surgery compared with routine care in high-risk patients having elective major open abdominal surgery.

**Methods:**

In three German and two Spanish centres, high-risk patients having elective major open abdominal surgery were randomised to cardiac index-guided therapy to maintain optimised postinduction cardiac index (cardiac index at which pulse pressure variation was <12%) during and for the first 8 h after surgery using intravenous fluids and dobutamine or to routine care. The primary outcome was the incidence of a composite outcome of moderate or severe complications within 28 days after surgery.

**Results:**

We analysed 318 of 380 enrolled subjects. The composite primary outcome occurred in 84 of 152 subjects (55%) assigned to cardiac index-guided therapy and in 77 of 166 subjects (46%) assigned to routine care (odds ratio: 1.87, 95% confidence interval: 1.03–3.39, *P*=0.038). Per-protocol analyses confirmed the results of the primary outcome analysis.

**Conclusions:**

Maintaining optimised postinduction cardiac index during and for the first 8 h after surgery did not reduce, and possibly increased, the incidence of a composite outcome of complications within 28 days after surgery compared with routine care in high-risk patients having elective major open abdominal surgery. Clinicians should not strive to maintain optimised postinduction cardiac index during and after surgery in expectation of reducing complications.

**Clinical trial registration:**

NCT03021525.

**Editor's key points**

• Optimising perfusion during surgery could help reduce postoperative complications.
• This multicentre randomised trial tested the hypothesis that maintaining optimised postinduction cardiac index during and after surgery reduces the incidence of complications within 28 days after surgery compared with routine care.
• Maintaining optimised postinduction cardiac index did not reduce, and possibly increased, the incidence of complications compared with routine care in high-risk patients having elective major open abdominal surgery.

About one in five patients having inpatient noncardiac surgery develops postoperative complications.^1^^,^^2^ Postoperative complications are associated with short-term^3^ and long-term^2^^,^^4^ postoperative mortality. Reducing postoperative complications might thus improve long-term surgical outcomes. The aetiology of postoperative complications is multifactorial but likely includes inadequate organ perfusion during surgery.^5^ Optimising blood flow (i.e. stroke volume or cardiac output) during surgery could thus help reduce postoperative complications,^6^, ^7^, ^8^ but optimal target values remain elusive.

Cardiac output is highly variable, both within and between individuals.^9^^,^^10^ We thus assumed that intraoperative cardiac output targets can best be individually defined after the induction of general anaesthesia.^11^ As cardiac output depends on intravascular fluid status, we further assumed that postinduction cardiac output is optimal when patients are unlikely to be fluid responsive. Fluid responsiveness, defined by an increase in cardiac output after fluid administration, can be predicted by pulse pressure variation.^12^, ^13^, ^14^ We therefore used pulse pressure variation to optimise postinduction cardiac index (cardiac output indexed to body surface area), and aimed to determine whether maintaining optimised postinduction cardiac index during surgery improves patient outcomes. Specifically, we tested the primary hypothesis that maintaining optimised postinduction cardiac index during and for the first 8 h after surgery reduces the incidence of a composite outcome of complications within 28 days after surgery compared with routine care in high-risk patients having elective major open abdominal surgery.

## Methods

### Trial design

The multicentre randomised ‘Individualized Perioperative Hemodynamic Goal-directed Therapy in Major Abdominal Surgery’ (iPEGASUS) trial was conducted between August 29, 2017 and August 26, 2023 in three German and two Spanish medical centres.^15^ The trial was approved by the primary ethics committee (Ethics Committee of the University of Gießen, Gießen, Germany) on February 8, 2017 (reference number 257/16) and by the ethics committees of all participating centres. All subjects provided written informed consent.

The trial was registered at ClinicalTrials.gov (NCT03021525) on January 16, 2017 and supervised by a data safety monitoring board. The statistical analysis plan was written and approved by the principal investigators and trial statisticians before data analysis. This trial is reported according to the Consolidated Standards of Reporting Trials statement.^16^

### Subjects

We enrolled consenting adults scheduled for elective major open abdominal (i.e. visceral, urological, or gynaecological) surgery expected to last >2 h whom we expected to get >2 L of intravenous fluids and who had ≥10% risk of postoperative complications according to the American College of Surgery – National Surgical Quality Improvement Program risk calculator.^17^ We excluded patients in whom laparoscopic surgery was planned; pregnant women; patients who had heart rhythms other than sinus rhythm, left ventricular ejection fraction <30%, aortic valve stenosis with an aortic valve area <1 cm^2^ or mean gradient >40 mm Hg, pheochromocytoma, acute myocardial ischaemia within 30 days before randomisation, septic shock, anuric renal failure; and patients receiving palliative treatment. Inclusion was further restricted to patients in whom clinicians did not plan to use cardiac index monitoring. We excluded patients after randomisation who had surgery lasting <2 h or who were given <2 L of intravenous fluids.

### Randomisation and protocol

We previously published the trial protocol.^15^ Basic anaesthetic management was per local routine. General anaesthesia was maintained with inhaled or intravenous anaesthetics. When clinically indicated, an epidural catheter was inserted before induction of general anaesthesia. Clinicians aimed to maintain mean arterial pressure >65 mm Hg, heart rate <100 beats min^−1^, peripheral oxygen saturation >93% (by varying inspired oxygen fraction, tidal volumes within 6–8 ml kg^−1^, and positive end-expiratory pressure 0–10 cm H_2_O), and body core temperature >36°C.

Subjects were centrally randomised to cardiac index-guided therapy to maintain optimised postinduction cardiac index during and for the first 8 h after surgery (cardiac index-guided therapy) or to routine care in a 1:1 ratio using computer-generated codes.

**Fig 1:**
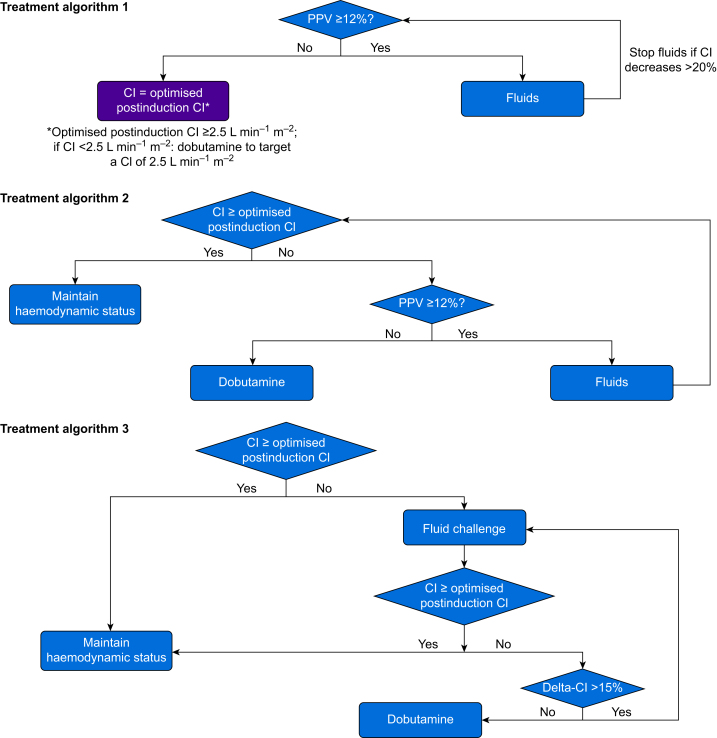
Treatment algorithms. Treatment algorithm 1 illustrates determination of the optimised postinduction cardiac index at which pulse pressure variation was <12% after induction of general anaesthesia. Clinicians were asked to maintain optimised postinduction cardiac index during surgery according to treatment algorithm 2. During the first 8 h after surgery, cardiac index-guided therapy was performed according to treatment algorithm 2 in subjects on controlled mechanical ventilation or according to treatment algorithm 3 in spontaneously breathing subjects. CI, cardiac index; PPV, pulse pressure variation.

In subjects assigned to cardiac index-guided therapy, cardiac index and pulse pressure variation were measured using internally calibrated pulse wave analysis (ProAQT; Pulsion Medical Systems, Feldkirchen, Germany).^18^, ^19^, ^20^ We assumed that postinduction cardiac index was optimal when pulse pressure variation was <12% (treatment algorithm 1, Fig. 1). If pulse pressure variation was ≥12% after the induction of general anaesthesia, we gave fluids until pulse pressure variation was <12%, and then defined the respective cardiac index as ‘optimised postinduction cardiac index’. If pulse pressure variation was <12% after induction of general anaesthesia, we defined the respective cardiac index as ‘optimised postinduction cardiac index’. In either case, the minimum ‘optimised postinduction cardiac index’ we allowed was 2.5 L min^−1^ m^−2^.

Clinicians were asked to maintain optimised postinduction cardiac index by giving dobutamine or 500 ml fluid boluses during surgery (treatment algorithm 2; Fig. 1), and, as practical, for the first 8 h after surgery. During the first 8 h after surgery, cardiac index-guided therapy was performed according to treatment algorithm 2 in subjects on controlled mechanical ventilation or according to treatment algorithm 3 in spontaneously breathing subjects (Fig. 1). In subjects assigned to routine care, clinicians managed haemodynamics without cardiac index monitoring per local clinical routine. Subjects were blinded to group allocation before and during surgery. Clinicians and outcome assessors were not blinded to group allocation.

### Outcomes

The primary outcome was the incidence of a collapsed (any event *vs* none) composite outcome of complications within 28 days after surgery (Supplementary Methods). Complications included death and 22 complications as defined by the European Perioperative Clinical Outcome definitions.^21^ We only considered complications if classified as moderate or severe.^21^

Secondary outcomes included the incidences of the composite outcome within 3 and 7 days after surgery; the individual incidences of each complication within 3, 7, and 28 days after surgery; the number of complications per subject within 3, 7, and 28 days after surgery; length of stay in the intensive care unit and in the hospital within 6 months after surgery; days alive within 6 months after surgery; and the incidence of death within 6 months after surgery.

Subjects were followed during their hospital stay and up to 6 months after surgery. Postoperative outcomes were assessed by reviewing electronic medical records and telephone interviews.

### Statistical analysis

Categorical data are presented as absolute number (percentage). Continuous data are presented as mean (standard deviation [sd]) or median (25th percentile–75th percentile). All statistical tests were performed two-sided at a 5% significance level. Confidence intervals (CIs) are presented as 95% two-sided. We calculated absolute standardised differences^22^ to describe baseline imbalances between the two treatment groups, and considered factors with absolute standardised differences >0.2 as imbalanced. The primary and secondary outcomes were assessed in the modified intention-to-treat population, that is subjects who were randomised, had surgery lasting >120 min, and who were given >2 L of fluid.

The incidence of the composite primary outcome was compared between subjects assigned to cardiac index-guided therapy and subjects assigned to routine care using a binary logistic regression model with treatment group and centre as independent variables. A treatment-by-centre interaction was also included in the model. We report adjusted odds ratios with 95% CIs and *P*-values.

As a sensitivity analysis, the analysis was conducted adjusting for imbalanced baseline variables between groups. In two separate per-protocol analyses, we compared the incidence of the primary outcome between subjects assigned to routine care and subjects assigned to cardiac index-guided therapy in whom: (a) cardiac index was at or above the optimised postinduction cardiac index for ≥80% of the intervention period (i.e. during surgery and during the first 8 h after surgery); and (b) treatment algorithm 2 was correctly followed for ≥80% of the time during surgery (Supplementary Methods).

Secondary outcomes were not adjusted for multiple analyses. Binary secondary outcomes are described using absolute and relative frequencies. The incidences of binary secondary outcomes were compared between subjects assigned to cardiac index-guided therapy and subjects assigned to routine care as for the primary outcome. For the individual incidences of each complication, statistical tests were restricted to complications within 28 days after surgery (owing to multiplicity) and to complications with at least two events per treatment group (owing to model computability). The other binary secondary outcomes are presented descriptively.

Count secondary outcomes were analysed using negative binomial regression models with treatment group and centre as independent variables. Analyses were restricted to complications with at least two events. The interaction term was kept in the model if the *P*-value for treatment-by-centre interaction was <0.05. Observed and adjusted event rates for the treatment groups, and the adjusted incidence rate ratios with 95% CI and *P-*values for the group comparison, are presented either overall or separated by centre when interaction terms were kept in the model.

The secondary outcome ‘days alive within 6 months after surgery’ was analysed using a linear regression model with treatment group and centre as independent variables. The interaction term was kept in the model if the *P*-value for treatment-by-centre interaction was <0.05. Observed mean (sd) for the treatment groups, and the adjusted mean difference with 95% CI and *P*-value for the group comparison, are presented either overall or separated by centre when interaction terms were kept in the model.

Analyses were performed using R version 4.2.2 (R Foundation for Statistical Computing, Vienna, Austria) or Stata 17 (StataCorp LLC, College Station, TX, USA).

### Sample size estimate

We assumed that the composite primary outcome would occur in 48% of subjects assigned to routine care. 167 subjects per group (*n*=334 in total) would provide 80% power for detecting an absolute reduction in the incidence of the composite primary outcome from 48% in subjects assigned to routine care to 33% in subjects assigned to cardiac index-guided therapy at a significance level of 5%. We planned to exclude the first two subjects in each centre *a priori* and to perform an interim analysis after recruitment of 50% of subjects. Allowing for 10% of subjects dropping out and excluding the first two subjects in each centre, we planned to enrol a total of 380 subjects (190 subjects per group).

## Results

### Subject enrolment and follow-up

**Fig 2:**
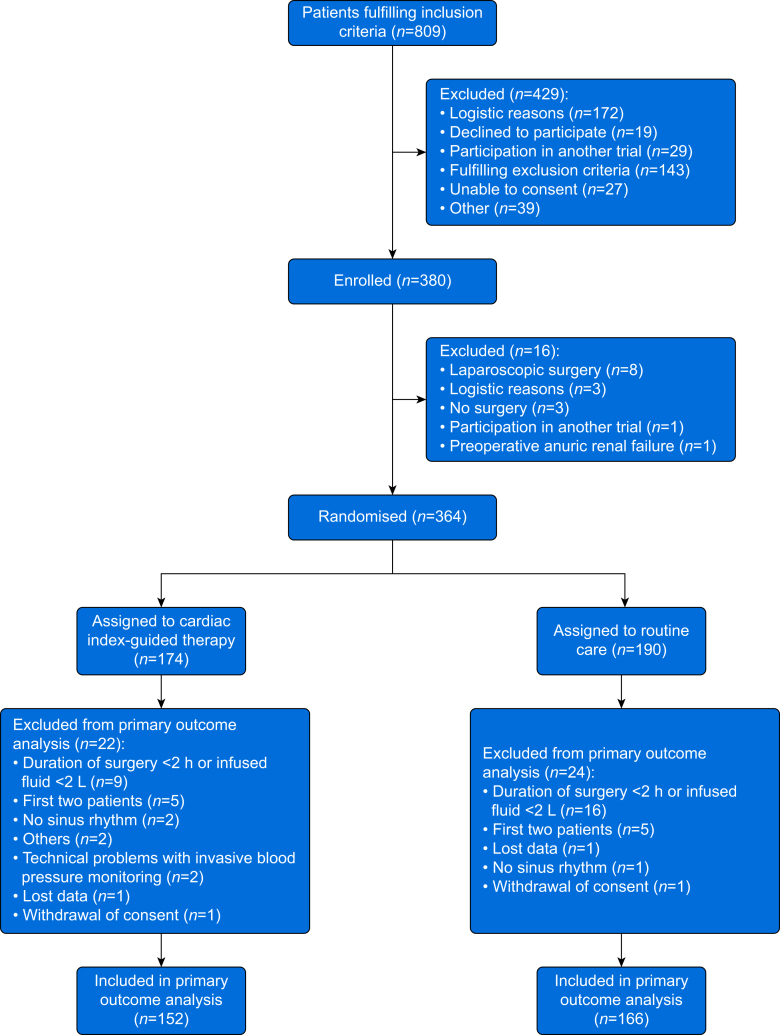
Subject flow chart. Flow chart illustrating subject screening, enrolment, randomisation, and reasons for exclusion.

As planned, we enrolled 380 subjects but excluded 16 before and 46 after randomisation (Fig. 2; Supplementary Table S2). We thus finally analysed 318 subjects, 152 (48%) assigned to cardiac index-guided therapy and 166 (52%) assigned to routine care. Demographic and baseline characteristics were generally well-balanced between the two groups, but subjects assigned to cardiac index-guided therapy had a higher body weight and were more often receiving chronic antihypertensive medications (Table 1). All subjects were followed up until 6 months after surgery.

**Table 1:** Baseline subject characteristics. Categorical data are presented as *n* (%), and continuous data are presented as median (25th percentile–75th percentile). Percentages may not sum up to 100% because of rounding. ACS-NSQUIP, American College of Surgery – National Surgical Quality Improvement Program.

Characteristic	Routine care (*n*=166)	Cardiac index-guided therapy (*n*=152)	Absolute standardised difference
**Age (yr)**	64 (55–75)	68 (61–74)	0.197
**Height (cm)**	171 (164–176)	170 (162–177)	0.097
**Weight (kg)**	73 (62–83)	78 (64–89)	0.206
**Sex, *n* (%)**			
Female	67 (40)	60 (39)	0.018
Male	99 (60)	92 (61)	
**American Society of Anesthesiologists physical status, *n* (%)**			
1	5 (3)	2 (1)	0.117
2	74 (45)	67 (44)	0.010
3	87 (52)	81 (53)	0.018
4	0 (0)	2 (1)	0.162
**ACS-NSQUIP risk score for serious postoperative complications (%)**	18 (13–25)	19 (14–26)	0.048
**ACS-NSQUIP risk score for any postoperative complications (%)**	22 (15–29)	22 (17–30)	0.068
**Baseline risk factors, *n* (%)**			
Chronic arterial hypertension/peripheral arterial occlusive disease	82 (49)	90 (59)	0.198
Chronic kidney disease	16 (10)	11(7)	0.087
Chronic obstructive pulmonary disease/chronic restrictive pulmonary disease	23 (14)	23 (15)	0.034
Diabetes mellitus	43 (26)	33 (22)	0.099
Reduced ejection fraction/right ventricular dysfunction/coronary artery disease	10 (6)	9 (6)	0.004
Stroke	6 (4)	8 (5)	0.083
Thrombosis/pulmonary artery embolism/coagulation disorder	10 (6)	8 (5)	0.030
Antihypertensive medication	75 (45)	90 (59)	0.283
Immunosuppressive medication	8 (5)	5 (3)	0.076
**Type of surgery, *n* (%)**			
Visceral	115 (69)	98 (64)	0.102
Urological	37 (22)	46 (30)	0.182
Gynaecological	14 (8)	8 (5)	0.123

### Trial treatment

In the 152 subjects assigned to cardiac index-guided therapy, the median optimised postinduction cardiac index was 2.6 (2.5–2.8) L min^−1^ m^−2^ and ranged from 2.5 L min^−1^ m^−2^ to 4.2 L min^−1^ m^−2^; 71 of these 152 (47%) subjects required fluids to achieve the optimised postinduction cardiac index (i.e. the cardiac index at which pulse pressure variation was <12%). The median amount of fluid subjects were given to achieve the optimised postinduction cardiac index was 500 (500–500) ml.

A vasopressor was given to 135 of the 152 subjects (89%) assigned to cardiac index-guided therapy and to 141 of the 166 subjects (85%) assigned to routine care. Subjects assigned to cardiac index-guided therapy were given slightly more fluids than subjects assigned to routine care (median total amount of fluids: 4380 [3557–5789] ml *vs* 4000 [3200–5263] ml; Table 2). Dobutamine was given to 66 of the 152 subjects (43%) assigned to cardiac index-guided therapy, but to none of the subjects assigned to routine care.

**Table 2:** Clinical characteristics. Categorical data are presented as *n* (%), and continuous data are presented as median (25th percentile–75th percentile).

Characteristic	Routine care (*n*=166)	Cardiac index-guided therapy (*n*=152)
**Duration**		
Duration of total intervention period (min)	690 (622–780)	719 (631–780)
Duration of surgery (min)	230 (180–300)	240 (180–321)
Duration of postoperative intervention period (h)	8.0 (7.7–8.0)	8.0 (8.0–8.0)
**Anaesthetic technique, *n* (%)**		
Balanced anaesthesia	122 (73)	120 (79)
Total intravenous anaesthesia	44 (27)	32 (21)
Epidural catheter	124 (75)	105 (69)
**Arterial blood pressure**		
Intra-arterial blood pressure monitoring, *n* (%)	141 (85)	152 (100)
Mean arterial pressure during surgery (mm Hg)	77 (73–82)	77 (72–83)
Mean arterial pressure during postoperative intervention period (mm Hg)	78 (72–84)	78 (72–86)
**Fluids**		
Crystalloids (ml)	3375 (2814–4199)	3732 (3032–4642)
Colloids (ml)	500 (0–700)	500 (0–1000)
Transfusion of fresh frozen plasma, *n* (%)	21 (13)	13 (9)
Transfusion of packed red cells, *n* (%)	32 (19)	36 (24)
Total amount of fluids (ml)	4000 (3200–5301)	4337 (3519–5804)
Amount of fluids during surgery (ml)	2700 (2200–4000)	3005 (2362–4300)
Amount of fluids during postoperative intervention period (ml)	1199 (700–1540)	1179 (797–1865)
**Vasopressor use, *n* (%)**		
Any vasopressor	141 (85)	135 (89)
Norepinephrine	99 (60)	119 (78)
Cafedrine/theodrenaline	44 (27)	39 (26)
Ephedrine	29 (17)	20 (13)
Phenylephrine	28 (17)	16 (11)
**Dobutamine use**		
Dobutamine, *n* (%)	0 (0)	66 (43)
Total dobutamine dose (*n*=66; mg)	–	27 (14–50)
Dobutamine dose during surgery (*n*=66; mg)	–	21 (12–47)
Dobutamine dose during postoperative intervention period (*n*=23; mg)	–	5 (2–49)
**Estimated blood loss (ml)**	500 (300–1000)	600 (400–950)
**Diuresis (ml)**	848 (570–1260)	908 (553–1270)
**Extubation after end of surgery, *n* (%)**	141 (85)	129 (85)
**Postoperative destination, *n* (%)**		
General ward	73 (44)	60 (39)
Intensive care or high-dependency unit	93 (56)	92 (61)

In 120 of the 152 subjects (79%) assigned to cardiac index-guided therapy, cardiac index was at or above the optimised postinduction cardiac index for ≥80% of the intervention period. In 143 of the 152 subjects (94%), treatment algorithm 2 was correctly followed ≥80% of the time during surgery. Cardiac index-guided therapy was continued until 8 h after surgery in 122 of the 152 subjects (80%) assigned to cardiac index-guided therapy (Supplementary Fig. S1).

### Primary outcome

**Fig 3:**
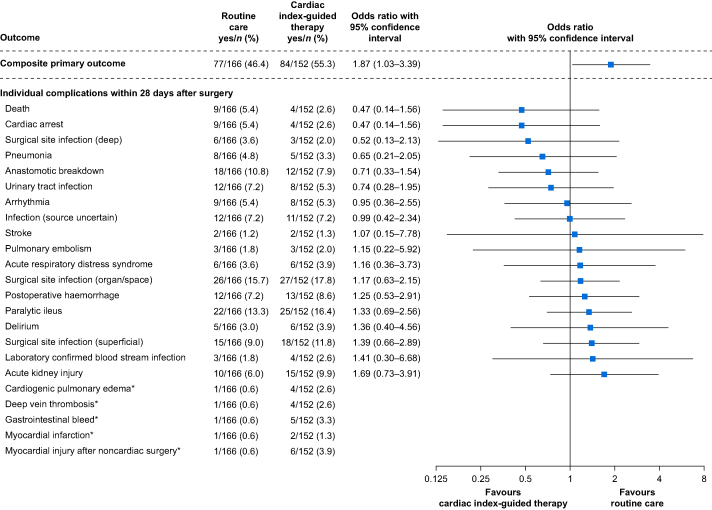
Primary outcome. Forest plots showing the effect of cardiac index-guided therapy compared with routine care on the composite primary outcome and individual complications within 28 days after surgery. ∗Statistical tests were restricted to complications with at least two events per treatment group owing to model computability.

The composite primary outcome occurred in 84 of 152 subjects (55%) assigned to cardiac index-guided therapy and in 77 of 166 subjects (46%) assigned to routine care (odds ratio: 1.87, 95% CI: 1.03–3.39, *P*=0.038; Fig. 3; Supplementary Fig. S2). Adjusting for imbalanced baseline variables (body weight and chronic antihypertensive medications) resulted in an adjusted odds ratio of 1.84 (95% CI: 1.01–3.38, *P*=0.047).

Per-protocol analyses confirmed the results of the primary outcome analysis in the modified intention-to-treat population. The composite primary outcome occurred in 67 of 120 subjects (56%) assigned to cardiac index-guided therapy in whom cardiac index was at or above the optimised postinduction cardiac index ≥80% of the intervention period (odds ratio: 1.79, 95% CI: 0.97–3.30, *P*=0.062) and in 79 of 143 subjects (55%) assigned to cardiac index-guided therapy in whom treatment algorithm 2 was correctly followed ≥80% of the time during surgery (odds ratio: 1.86, 95% CI: 1.03–3.39, *P*=0.041).

### Secondary outcomes

The individual incidences of each complication within 28 days after surgery are shown in Fig. 3. The composite outcome occurred more frequently in subjects assigned to cardiac index-guided therapy than in subjects assigned to routine care both within 3 days after surgery (22% *vs* 17%) and within 7 days after surgery (37% *vs* 28%) (Supplementary Table S1). Subjects assigned to cardiac index-guided therapy had more complications within 3, 7, and 28 days after surgery than subjects assigned to routine care (Supplementary Table S1). At 6 months after surgery, 10 of the 152 subjects (7%) assigned to cardiac index-guided therapy and 21 of the 166 subjects (13%) assigned to routine care had died (odds ratio: 0.48, 95% CI: 0.22–1.05, *P*=0.065; Table 3; Supplementary Fig. S3).

**Table 3:** Secondary outcomes. Categorical data are presented as *n* (%) with odds ratio (95% confidence interval [CI]). Continuous data are presented as mean (standard deviation) with incidence rate ratio (95% CI) or mean difference (95% CI). The *P*-value for treatment-by-centre interaction was >0.05 for all presented outcomes (except for ‘length of stay in the intensive care unit within 6 months after surgery’ [*P*<0.001] and ‘length of stay in the hospital within 6 months after surgery’ [*P*=0.009]). Centre 1: University Medical Center Hamburg-Eppendorf, Hamburg, Germany; Centre 2: University Medical Centre of Rostock, Rostock, Germany; Centre 3: Justus-Liebig-University Giessen, Giessen, Germany; Centre 4: Hospital de la Santa Creu i Sant Pau, Barcelona, Spain; Centre 5: Hospital Universitari i Politécnic La Fe, Valencia, Spain. ∗The effect size is an incidence rate ratio. ^†^The effect size is a mean difference. ^‡^The effect size is an odds ratio.

Outcome		Routine care (*n*=166)	Cardiac index-guided therapy (*n*=152)	Effect size	*P*-value
**Number of complications per subject within 28 days after surgery, n/*n***		1.1 (1.8)	1.3 (1.8)	1.13∗ (0.82–1.57)	0.447
**Length of stay in the intensive care unit within 6 months after surgery (days)**	Centre 1	1.8 (2.0)	4.0 (7.4)	2.24∗ (0.80–6.24)	0.123
	Centre 2	1.9 (2.5)	5.5 (7.4)	2.82∗ (1.22–6.53)	0.016
	Centre 3	2.9 (6.4)	2.7 (4.0)	0.91∗ (0.58–1.43)	0.697
	Centre 4	4.8 (12.6)	2.3 (3.5)	0.48∗ (0.26–0.87)	0.016
	Centre 5	0.5 (0.9)	2.3 (5.2)	4.64∗ (2.13–10.13)	<0.001
**Length of stay in the hospital within 6 months after surgery (days)**	Centre 1	22.6 (15.2)	18.5 (17.4)	0.82∗ (0.46–1.46)	0.497
	Centre 2	24.0 (14.5)	43.3 (45.0)	1.80∗ (1.12–2.90)	0.015
	Centre 3	17.9 (13.4)	15.7 (12.03)	0.88∗ (0.68–1.14)	0.319
	Centre 4	23.7 (23.3)	28.2 (25.5)	1.19∗ (0.85–1.68)	0.312
	Centre 5	14.4 (16.1)	24.6 (26.1)	1.71∗ (1.19–2.45)	0.004
**Days alive within 6 months after surgery (days)**		167.2 (44.2)	174.1 (33.9)	7.10^†^ (-1.68–15.87)	0.113
**Death within 6 months after surgery, *n* (%)**		21 (13)	10 (7)	0.48^‡^ (0.22–1.05)	0.065

## Discussion

Contrary to our hypothesis, maintaining optimised postinduction cardiac index during and for the first 8 h after surgery did not reduce, and possibly increased, the incidence of a composite outcome of complications within 28 days after surgery compared with routine care in high-risk patients having elective major open abdominal surgery.

There are numerous previous trials on cardiac output-guided management in patients having noncardiac surgery.^6^, ^7^, ^8^^,^^23^ It nevertheless remains controversial whether targeted perioperative cardiac output management improves postoperative outcomes.^8^ The results of most multicentre trials are consistent with ours, and indicate that cardiac output-guided management does not improve outcomes in patients having noncardiac surgery. In a multicentre trial including 482 abdominal surgery patients, targeting fixed age-specific cardiac index values did not reduce major complications compared with routine care.^24^ In the two largest trials so far, maximising stroke volume with fluid challenges after anaesthetic induction and maintaining this maximised postinduction stroke volume by giving fluids and a fixed low dose of an inotrope during major gastrointestinal surgery did not reduce the incidence of a composite outcome of 30-day postoperative complications and death in 734 patients (OPTIMISE^25^) or the incidence of 30-day postoperative infectious complications in 2498 patients (OPTIMISE II^26^; results presented at the EBPOM World Congress of Prehabilitation Medicine 2023 in London on July 6, 2023).

We assumed that postinduction cardiac index is optimal when patients are unlikely to be fluid responsive. We thus used pulse pressure variation to optimise postinduction cardiac index, and then maintained optimised cardiac index during and for the first 8 h after surgery. We chose to define optimised cardiac index after induction of general anaesthesia for two main reasons. Firstly, cardiac index is mainly determined by metabolic needs.^27^ Energy expenditure after induction of general anaesthesia is about 25% lower than awake resting energy expenditure and reflects energy expenditure during abdominal surgery.^28^ Secondly, defining cardiac index targets after induction of general anaesthesia allowed us to use pulse pressure variation that can predict fluid responsiveness only during controlled mechanical ventilation.^13^^,^^14^^,^^29^

Protocol adherence is crucial in trials of targeted haemodynamic management. Adherence was good in our trial. Cardiac index was sustained at or above the optimised postinduction cardiac index for ≥80% of the intervention period in 79% of subjects, and the treatment algorithm was correctly followed for ≥80% of the time during surgery in 94% of subjects. It is thus highly unlikely that better protocol adherence would have changed our findings. The question, then, is whether a different protocol might have produced favourable outcomes.

Using optimised postinduction cardiac index as intraoperative target might not have been the best approach. Anaesthetic induction agents can produce myocardial depression and vasodilation resulting in relative hypovolaemia.^30^, ^31^, ^32^ Cardiac index measured just after induction of general anaesthesia might thus be particularly low and pulse pressure variation particularly high. Consistent with this, about half of the subjects required fluids to achieve the optimised postinduction cardiac index (i.e. to decrease pulse pressure variation to <12%).

Targeting cardiac index measured *before* induction of anaesthesia might have been preferable intraoperatively, and likely would have been preferable postoperatively. Consistent with this assumption, maintaining preoperative resting cardiac index reduced major postoperative complications within 30 days after surgery compared with routine care in 188 high-risk patients having major abdominal surgery.^33^ Additionally, as fluid responsiveness is not the same as the need for fluids, it might be better to simply define cardiac index targets without considering fluid responsiveness.

Our results suggest that maintaining optimised postinduction cardiac index might increase the risk for postoperative complications, but potential underlying mechanisms remain speculative. The median total amount of fluids was only slightly higher in subjects assigned to cardiac index-guided therapy than in subjects assigned to routine care, and > 80% of subjects in each group were given vasopressors. Aside from potential differences in fluid timing, use of dobutamine was a main difference in the haemodynamic management between subjects assigned to cardiac index-guided therapy and subjects assigned to routine care. It remains unclear whether use of dobutamine was directly linked to complications.

A limitation of our trial is that recruiting 380 subjects took 6 yr, in part because of the COVID-19 pandemic. Furthermore, we performed the trial in only five centres. Including more centres would presumably have sped up recruitment and improved external validity. Additionally, subjects were blinded to group allocation before and during surgery, but could have recognised group allocation in the postoperative period. Outcome assessors were not systematically blinded to group allocation. However, it is unlikely that not blinding outcome assessors introduced bias because we used objective outcomes and monitored outcome data. We measured cardiac index using internally calibrated pulse wave analysis, which can become unreliable when vasomotor tone is markedly altered or rapidly changes.^18^, ^19^, ^20^ However, the specific pulse wave analysis system we used is validated in surgical patients.^34^ Additionally, using pulse wave analysis reflects current clinical practice because it is the most commonly used method to measure cardiac index during surgery.^35^^,^^36^ Finally, we only measured cardiac index in subjects assigned to cardiac index-guided therapy, but not in subjects assigned to routine care. Blinded cardiac index monitoring in subjects assigned to routine care would have allowed to determine if intraoperative cardiac index markedly differed between treatment groups.

In conclusion, maintaining optimised postinduction cardiac index during and for the first 8 h after surgery did not reduce, and possibly increased, the incidence of a composite outcome of complications within 28 days after surgery compared with routine care in high-risk patients having elective major open abdominal surgery. Clinicians should not strive to maintain optimised postinduction cardiac index during and after surgery in the expectation of reducing complications.

## Authors’ contributions

Trial conception and design: SF, SAH, DAR, BS

Data acquisition: GS, AB, PAN, GAC, SBE, ODC, FE, NGG, MH, GK, CK, AK, TM, VMG, AZ, MS, SAH

Data analysis and interpretation: all authors

Statistical analysis: SL, AP

Drafting of manuscript: SF, GS, AB, SL, DIS, DAR, BS

Critical revision of manuscript for important intellectual content: all authors

Final approval of the version to be published: all authors

Agreement to be accountable for all aspects of the work, thereby ensuring that questions related to the accuracy or integrity of any part of the work are appropriately investigated and resolved: all authors

## Declarations of interest

SF, GS, AB, PAN, GAC, SBE, FE, NGG, GK, CK, AK, TM, VMG, SL, AP, DIS, and SAH declare that they have no conflict of interest related to this trial. ODC has received research grants and funding for medical advice or transportation from MSD, Bbraun, Menarini, Masimo and/or Norgine. MH has received honoraria for giving lectures from Edwards Lifesciences (Irvine, CA, USA), and has received honoraria for giving lectures from Baxter (Deerfield, IL, USA). AZ has received a research grant, honoraria for giving lectures and refunds of travel expenses from Edwards Lifesciences (Irvine, CA, USA). MS is a consultant for Edwards Lifesciences and has received institutional research funding for investigator-initiated trials and honoraria for giving lectures from Edwards Lifesciences, has received honoraria for giving lectures from AMOMED (Vienna, Austria), Orion Pharma (Hamburg, Germany), and Philips Medizin Systeme Böblingen (Böblingen, Germany). DAR has received research funding (institutional) from Edwards and Gettinge, and has received speaker honoraria from Edwards, Baxter, Ratiopharm, and Philips. BS is a consultant for and has received institutional restricted research grants and honoraria for giving lectures from Edwards Lifesciences (Irvine, CA, USA). BS is a consultant for Philips North America (Cambridge, MA, USA) and has received honoraria for giving lectures from Philips Medizin Systeme Böblingen (Böblingen, Germany). BS has received institutional restricted research grants and honoraria for giving lectures from Baxter (Deerfield, IL, USA). BS is a consultant for and has received institutional restricted research grants and honoraria for giving lectures from GE Healthcare (Chicago, IL, USA). BS has received institutional restricted research grants and honoraria for giving lectures from CNSystems Medizintechnik (Graz, Austria). BS is a consultant for Maquet Critical Care (Solna, Sweden). BS has received honoraria for giving lectures from Getinge (Gothenburg, Sweden). BS is a consultant for and has received institutional restricted research grants and honoraria for giving lectures from Pulsion Medical Systems (Feldkirchen, Germany). BS is a consultant for and has received institutional restricted research grants and honoraria for giving lectures from Vygon (Aachen, Germany). BS is a consultant for and has received institutional restricted research grants from Retia Medical (Valhalla, NY, USA). BS has received institutional restricted research grants from Osypka Medical (Berlin, Germany). BS is a consultant for Dynocardia (Cambridge, MA, USA). BS was a consultant for and has received institutional restricted research grants from Tensys Medical (San Diego, CA, USA). BS is an editor of the *British Journal of Anaesthesia*.

## Funding

Pulsion Medical Systems, Germany and institutional and departmental sources.
