# Supplementary material for: Cardiac index-guided therapy to maintain optimised postinduction cardiac index in high-risk patients having major open abdominal surgery: the multicentre randomised iPEGASUS trial
Source: Br J Anaesth. 2024 May 26;133(2):277–87. doi: 10.1016/j.bja.2024.03.040 (PMC11282469; doi:10.1016/j.bja.2024.03.040)
Supplement: Multimedia component 1 [file mmc1.pdf]

Cardiac index-guided therapy to maintain optimised postinduction cardiac index in high-risk patients having major open abdominal surgery: the multicentre randomised iPEGASUS trial

Appendices

Contents

Supplementary Methods 2

Primary outcome 2

Per-protocol analysis b).. 2

Supplementary Figures 4

Supplementary Tables.. 7

Supplementary Methods

Primary outcome

The primary outcome was the incidence of a collapsed composite outcome of complications within 28 days after surgery. Complications included death and 22 complications according to European Perioperative Clinical Outcome definitions.[21] We only considered complications if classified as moderate or severe.[21] Specifically, the following 22 postoperative complications were considered: acute kidney injury, acute respiratory distress syndrome, anastomotic breakdown, arrhythmia, cardiac arrest, cardiogenic pulmonary oedema, deep vein thrombosis, delirium, gastrointestinal bleed, infection (source uncertain), laboratory confirmed blood stream infection, myocardial infarction, myocardial injury after non-cardiac surgery, pneumonia, paralytic ileus, postoperative haemorrhage, pulmonary embolism, stroke, surgical site infection (superficial), surgical site infection (deep), surgical site infection (organ/space), urinary tract infection.

Per-protocol analysis b)

We compared the incidence of the primary outcome between subjects assigned to routine care and subjects assigned to cardiac index-guided therapy in whom treatment algorithm 2 was correctly followed for ≥80% of the time during surgery.

Treatment according to the treatment algorithm 2 was defined as follows: If the cardiac index was higher than or equal to the optimised postinduction cardiac index, subjects were considered to be treated according to the treatment algorithm 2 for the next 30 minutes. If the cardiac index was below the optimised postinduction cardiac index and the pulse pressure variation was higher than or equal to 12% and subjects were given at least 500 ml fluids during the next 30 minutes, subjects were considered to be treated according to the treatment algorithm 2 for the next 30 minutes. If the

cardiac index was below the optimised postinduction cardiac index and the pulse pressure variation was below 12% and subjects were given dobutamine during the next 30 minutes, subjects were considered to be treated according to the treatment algorithm 2 for the next 30 minutes. In all other cases, subjects were considered not to be treated according to the treatment algorithm 2 for the next 30 minutes.

Supplementary Figures

Supplementary Fig. S1: Histogram of postoperative intervention period durations

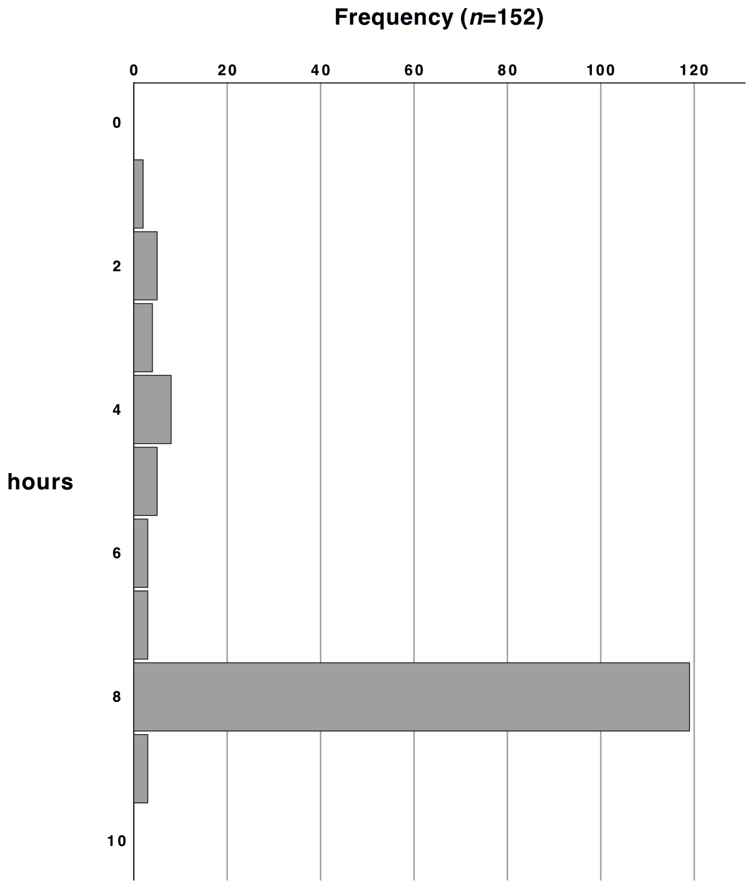

Histograms showing the distribution of frequencies of postoperative intervention period durations for subjects assigned to cardiac index-guided therapy.

Supplementary Fig. S2: Primary outcome separated by centre

Centre	Routine care yes/n (%)	Cardiac index-guided therapy yes/n (%)	Odds ratio with 95% confidence interval
1	10/14 (71.4)	8/10 (80.0)	1.60 (0.23 - 11.1)
2	7/17 (41.2)	14/17 (82.4)	6.67 (1.38 - 32.3)
3	24/64 (37.5)	24/62 (38.7)	1.05 (0.51 - 2.2)
4	24/35 (68.6)	24/34 (70.6)	1.10 (0.39 - 3.1)
5	12/36 (33.3)	14/29 (48.3)	1.87 (0.68 - 5.1)
**Overall**	77/166 (46.4)	84/152 (55.3)	1.87 (1.03 - 3.4)

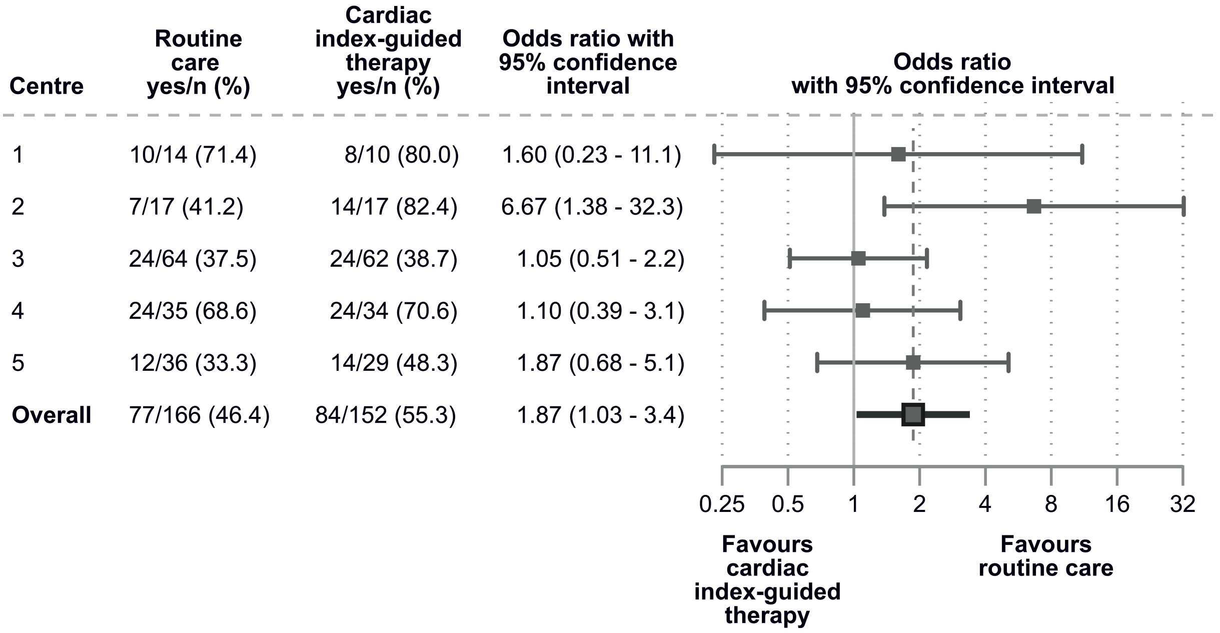

Forest plot showing the effect of cardiac index-guided therapy compared with routine care on the composite primary outcome within 28 days after surgery separated by centre.

Centre 1: University Medical Center Hamburg-Eppendorf, Hamburg, Germany; Centre 2: University Medical Centre of Rostock, Rostock, Germany; Centre 3: Justus-Liebig-University Giessen, Giessen, Germany; Centre 4: Hospital de la Santa Creu i Sant Pau, Barcelona, Spain; Centre 5: Hospital Universitari i Politécnic La Fe, Valencia, Spain.

Supplementary Fig. S3: Kaplan-Meier curves

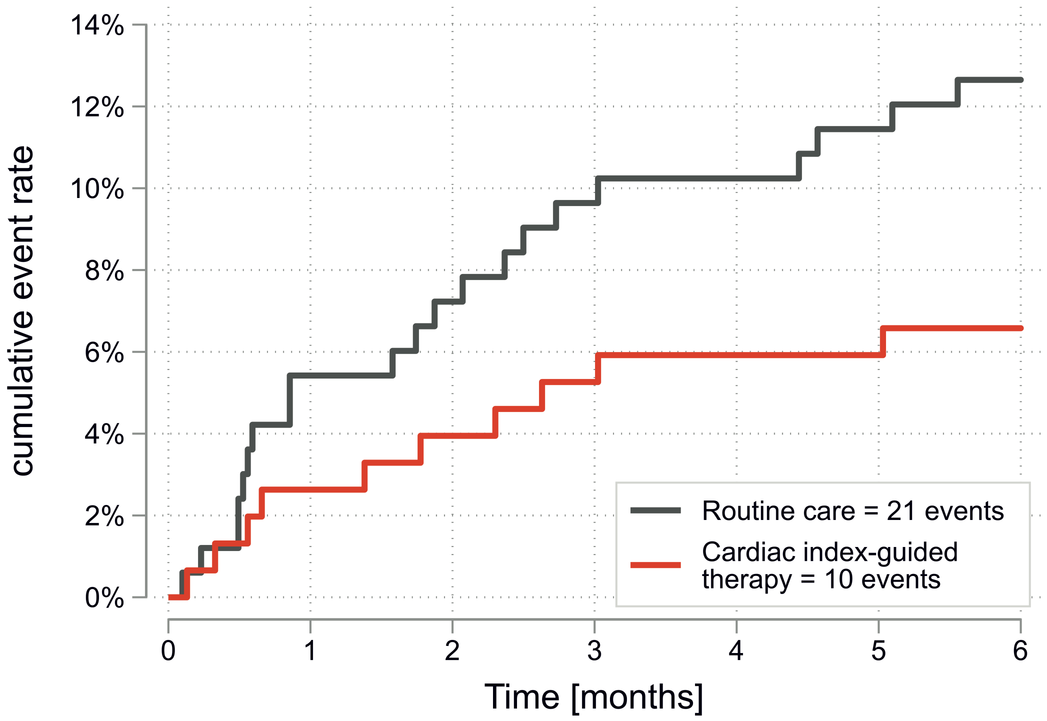

**Number at risk**							
Routine care	166	157	154	150	149	147	145
Cardiac index-guided therapy	152	148	146	144	143	143	142

Kaplan-Meier curves showing the cumulative event rates of death up to 6 months after surgery for the cardiac index-guided therapy group and routine care group.

Supplementary Tables

Supplementary Table S1: Additional secondary outcomes

**Outcome**	**Routine care (n=166)**	**Cardiac index-guided therapy (n=152)**	**Effect size**	**P value**
**Individual complications within 28 days after surgery**				
Acute kidney injury, n	10 (6%)	15 (10%)	1.69§ (0.73 – 3.91)	0.217
Acute respiratory distress syndrome, n	6 (4%)	6 (4%)	1.16§ (0.36 – 3.73)	0.799
Anastomotic breakdown, n	18 (11%)	12 (8%)	0.71§ (0.33, 1.54)	0.382
Arrhythmia, n	9 (5%)	8 (5%)	0.95§ (0.36 – 2.55)	0.923
Cardiac arrest, n	9 (5%)	4 (3%)	0.47§ (0.14 – 1.56)	0.216
Cardiogenic pulmonary oedema*, n	1 (1%)	4 (3%)		
Death, n	9 (5%)	4 (3%)	0.47§ (0.14 – 1.56)	0.216
Deep vein thrombosis*, n	1 (1%)	4 (3%)		
Delirium, n	5 (3%)	6 (4%)	1.36§ (0.40 – 4.56)	0.623
Gastrointestinal bleed*, n	1 (1%)	5 (3%)		
Infection (source uncertain) , n	12 (7%)	11 (7%)	0.99§ (0.42 – 2.34)	0.980
Laboratory confirmed blood stream infection, n	3 (2%)	4 (3%)	1.41§ (0.30 – 6.68)	0.666
Myocardial infarction*, n	1 (1%)	2 (1%)		
Myocardial injury after non-cardiac surgery*, n	1 (1%)	6 (4%)		
Pneumonia, n	8 (5%)	5 (3%)	0.65§ (0.21 – 2.05)	0.460
Paralytic ileus, n	22 (13%)	25 (16%)	1.33§ (0.69 – 2.56)	0.395
Postoperative haemorrhage, n	12 (7%)	13 (9%)	1.25§ (0.53 – 2.91)	0.612
Pulmonary embolism, n	3 (2%)	3 (2%)	1.15§ (0.22 – 5.92)	0.869
Stroke, n	2 (1%)	2 (1%)	1.07§ (0.15 – 7.78)	0.944

Outcome	Routine care (n=166)	Cardiac index-guided therapy (n=152)	Effect size	P value
Surgical site infection (superficial), n	15 (9%)	18 (12%)	1.39§ (0.66 – 2.89)	0.384
Surgical site infection (deep), n	6 (4%)	3 (2%)	0.52§ (0.13 – 2.13)	0.361
Surgical site infection (organ/space), n	26 (16%)	27 (18%)	1.17§ (0.63 – 2.15)	0.626
Urinary tract infection, n	12 (7%)	8 (5%)	0.74§ (0.28 – 1.95)	0.546
**Composite outcome within 3 days after surgery, n**	28 (17%)	34 (22%)	1.46§ (0.83 – 2.58)	0.193
**Composite outcome within 7 days after surgery, n**	47 (28%)	56 (37%)	1.56§ (0.95 – 2.56)	0.079
**Individual complications within 3 days after surgery***				
Acute kidney injury, n	4 (2%)	8 (5%)		
Acute respiratory distress syndrome, n	1 (1%)	3 (2%)		
Anastomotic breakdown, n	2 (1%)	3 (2%)		
Arrhythmia, n	4 (2%)	2 (1%)		
Cardiac arrest, n	1 (1%)	0 (0%)		
Cardiogenic pulmonary oedema, n	0 (0%)	0 (0%)		
Death, n	1 (1%)	0 (0%)		
Deep vein thrombosis, n	0 (0%)	2 (1%)		
Delirium, n	2 (1%)	5 (3%)		
Gastrointestinal bleed, n	1 (1%)	0 (0%)		
Infection (source uncertain), n	4 (2%)	3 (2%)		
Laboratory confirmed blood stream infection, n	1 (1%)	0 (0%)		
Myocardial infarction, n	1 (1%)	1 (1%)		
Myocardial injury after non-cardiac surgery, n	1 (1%)	5 (3%)		
Pneumonia, n	3 (2%)	2 (1%)		
Paralytic ileus, n	6 (4%)	4 (3%)		
Postoperative haemorrhage, n	9 (5%)	9 (6%)		

Outcome	Routine care (n=166)	Cardiac index-guided therapy (n=152)	Effect size	P value
Pulmonary embolism, n	0 (0%)	0 (0%)		
Stroke, n	1 (1%)	2 (1%)		
Surgical site infection (superficial), n	1 (1%)	2 (1%)		
Surgical site infection (deep), n	0 (0%)	0 (0%)		
Surgical site infection (organ/space), n	2 (1%)	2 (1%)		
Urinary tract infection, n	3 (2%)	0 (0%)		
**Individual complications within 7 days after surgery***				
Acute kidney injury, n	5 (3%)	10 (7%)		
Acute respiratory distress syndrome, n	3 (2%)	4 (3%)		
Anastomotic breakdown, n	7 (4%)	4 (3%)		
Arrhythmia, n	6 (4%)	5 (3%)		
Cardiac arrest, n	1 (1%)	0 (0%)		
Cardiogenic pulmonary oedema, n	0 (0%)	0 (0%)		
Death, n	1 (1%)	0 (0%)		
Deep vein thrombosis, n	1 (1%)	3 (2%)		
Delirium, n	4 (2%)	5 (3%)		
Gastrointestinal bleed, n	0 (0%)	2 (1%)		
Infection (source uncertain), n	9 (5%)	6 (4%)		
Laboratory confirmed blood stream infection, n	2 (1%)	1 (1%)		
Myocardial infarction, n	1 (1%)	1 (1%)		
Myocardial injury after non-cardiac surgery, n	1 (1%)	6 (4%)		
Pneumonia, n	6 (4%)	4 (3%)		
Paralytic ileus, n	17 (10%)	23 (15%)		
Postoperative haemorrhage, n	8 (5%)	10 (7%)		
Pulmonary embolism, n	1 (1%)	2 (1%)		
Stroke, n	1 (1%)	2 (1%)		

**Outcome**	**Routine care (n=166)**	**Cardiac index-guided therapy (n=152)**	**Effect size**	**P value**
Surgical site infection (superficial), n	6 (4%)	8 (5%)		
Surgical site infection (deep), n	0 (0%)	1 (1%)		
Surgical site infection (organ/space), n	7 (4%)	5 (3%)		
Urinary tract infection, n	6 (4%)	3 (2%)		
**Number of complications per subject within 3 days after surgery, n/n**	0.3 (0.8)	0.35 (0.8)	1.26# (0.73 – 2.17)	0.414
**Number of complications per subject within 7 days after surgery, n/n**	0.6 (1.1)	0.7 (1.2)	1.28# (0.84 – 1.95)	0.244
**Days free of mechanical ventilation within 28 days after surgery, days**	26.5 (5.1)	26.9 (4.6)	1.01# (0.97 – 1.06)	0.536
**Days free of vasopressor therapy within 28 days after surgery, days**	26.3 (4.6)	26.6 (4.0)	1.01# (0.97 – 1.05)	0.654
**Days free of renal replacement therapy within 28 days after surgery, days**	26.9 (4.4)	27.3 (3.5)	1.01# (0.97 – 1.06)	0.572

Categorical data are presented as number (percentage) with odds ratio (95%-confidence interval). Continuous data are presented as mean (standard deviation) with incidence rate ratio (95%-confidence interval). All calculated p values for treatment-by-centre interaction were >0.05.

*No statistical modelling was performed.

§The effect size is an odds ratio.

#The effect size is an incidence rate ratio.

Supplementary Table S2: Number of enrolled subjects per centre

Centre	Number of enrolled subjects, n
University Medical Center Hamburg-Eppendorf, Hamburg, Germany	32 (8%)
University Medical Centre of Rostock, Rostock, Germany	40 (11%)
Justus-Liebig-University Giessen, Giessen, Germany	151 (40%)
Hospital de la Santa Creu i Sant Pau, Barcelona, Spain	83 (22%)
Hospital Universitari i Politécnic La Fe, Valencia, Spain	74 (19%)

Categorical data are presented as number (percentage).
